# Effect of the Transition Points Mismatch on Quanta Image Sensors

**DOI:** 10.3390/s18124357

**Published:** 2018-12-10

**Authors:** Jiangtao Xu, Xiyang Zhao, Liqiang Han, Kaiming Nie, Liang Xu, Jianguo Ma

**Affiliations:** 1 School of Microelectronics, Tianjin University and Tianjin Key Laboratory of Imaging and Sensing Microelectronic Technology, No. 92 Weijin Road, Nankai District, Tianjin 300072, China; xujiangtao@tju.edu.cn (J.X.); seeyung@tju.edu.cn (X.Z.); nkaiming@tju.edu.cn (K.N.); xuliang323923@tju.edu.cn (L.X.); majg@tju.edu.cn (J.M.); 2 School of Electrical and Information Engineering, Tianjin University and Tianjin Key Laboratory of Imaging and Sensing Microelectronic Technology, No. 92 Weijin Road, Nankai District, Tianjin 300072, China

**Keywords:** ADC, bit error rate, imaging model, quanta image sensor, transition point

## Abstract

Mathematical models and imaging models that show the relationship between the transition points mismatch of analog-to-digital converters (ADCs) and the bit error rate (BER) in single-bit and multi-bit quanta image sensors (QISs) are established. The mathematical models suggest that when the root-mean-square (r.m.s.) of the read noise in jots is 0.15e^−^, the standard deviation of the transition points should be less than 0.15e^−^ to ensure that the BER is lower than 1% in the single-bit QIS, and 0.21e^−^ to ensure that the BER is lower than 5% in the multi-bit QIS. Based on the mathematical models, the imaging models prove that the fixed-pattern noise (FPN) increases with a stronger transition point mismatch. The imaging models also compare the imaging quality in the case of different spatial oversampling factors and bit depths. The grayscale similarity index (GSI) is 3.31 LSB and 1.74 LSB when the spatial oversampling factors are 256 and 4096, respectively, in the single-bit QIS. The GSI is 1.93 LSB and 1.13 LSB when the bit depth is 3 and 4, respectively, in the multi-bit QIS. It indicates that a higher bit depth and a larger spatial oversampling factor could reduce the effect of the transition points mismatch of1-bit or *n*-bit ADCs.

## 1. Introduction

As pixel shrink causes a drop in the number of photons detected by each pixel and a smaller full well capacity (FWC) that lead to a reduced signal-to-noise ratio and dynamic range, the quanta image sensor (QIS) has been proposed. The QIS was first introduced in 2005 [1], and the concept was clearly defined in 2011 [2]. A QIS can be achieved through single photon avalanche detectors [3,4] or active pixel sensors [5,6]. This paper concentrates on the active-pixel-based QIS. A QIS employs sub-diffraction-limit pixels with sub-electron read noise to achieve spatial oversampling and photon counting. The specialized pixel is referred to as “jot” [1]. A QIS can also achieve temporal oversampling through working at a high frame rate. Because of the characteristics of spatial and temporal oversampling and photon counting, QISs have a wide-ranging application space, which includes low-light imaging, high-speed imaging, and high dynamic range imaging [7,8,9]. The QIS concept includes the single-bit QIS and the multi-bit QIS, and their quantization processes are accomplished by 1-bit and *n*-bit analog-to-digital converters (ADCs), respectively.

The bit error rate (BER)—the probability of miscounting photoelectrons—is a key performance indicator to evaluate the imaging quality of a QIS [10]. It is influenced by read noise, the gain variation in jots, and the quanta exposure level, which have all been discussed in [11]. Our work is to concentrate on another important non-ideal characteristic: the transition points mismatch of 1-bit or *n*-bit ADCs. The influence of ADC transition points has been simply mentioned in [12], and this paper would go further and discuss the non-uniformity of ADC transition points. Transition points of fabricated ADCs always deviate from their design values, and, thus, the quantization process does not execute ideally. So, the relationship between the mismatch of ADC transition points and the BER needs to be investigated for further QIS design.

In fact, the voltage signal that needs to be resolved in a QIS is only a few hundred microvolts, so it is better to implement a pre-amplifier before the ADC no matter whether the QIS is single-bit or multi-bit [13]. A pre-amplifier suffers from an unavoidable input-referred offset that is caused by the device mismatch, so the transition points mismatch of well-calibrated ADC mainly comes from the input-referred offset of the pre-amplifier. In [14], a charge transfer amplifier is reported to have about a ±1 mV offset. It is good enough in conventional applications. However, due to the extremely small jot signal, a QIS has a stricter requirement for ADC transition points that result from the pre-amplifier. Bit errors caused by column [15] or cluster [5] parallel ADCs may lead to stripes or plaques in the final image.

This work exhibits BER as a function of ADC transition points mismatch. It would guide the design of the ADC in the future. The imaging model shows that the ADC design process for a QIS could be flexible according to the spatial and temporal oversampling factors.

The remainder of this paper is organized as follows. Section 2 presents mathematical models for the single-bit QIS and the multi-bit QIS to show the relationship between ADC transition points and the BER. Section 3 presents imaging models on the basis of the mathematical models. The imaging models are used to observe the imaging quality of a QIS intuitively. Section 4 concludes the paper.

## 2. The Transition Points Mismatch and Its Influence on the BER in a QIS

**Figure 1:**
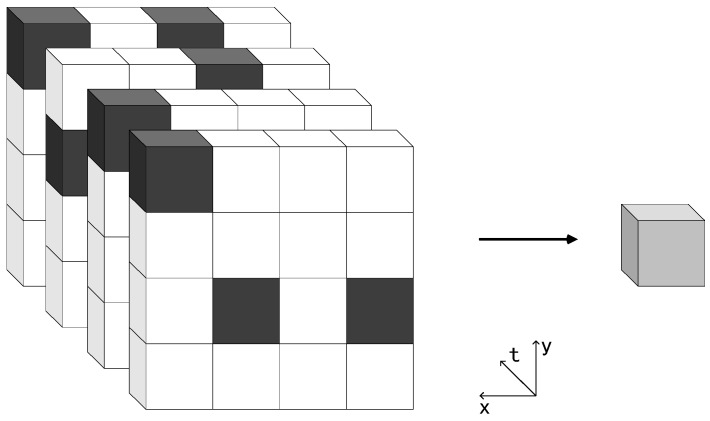
A 4 × 4 × 4 cubicle of jots bit data forms one pixel in the final image.

In a single-bit QIS, jots have two binary states: “0” means the jot collects no photon; “1” means the jot collects at least one photon. So, the jot array outputs a bit cube and each bit plane in it represents a captured field. One pixel in the final image is reconstructed from a small “cubicle” of bits from the jot data cube [16,17], and an example is shown in Figure 1. The cubicle has two spatial dimensions (x, y) and one temporal dimension (t). The spatial oversampling factor is the product of the x-axis and y-axis values, and the temporal oversampling factor is the t-axis value. In Figure 1, they are 16 and 4, respectively. In a multi-bit QIS, one jot outputs *n*-bit data in one frame, which represents the number of photoinduced electrons. The number *n* is the bit depth of the multi-bit QIS. 

In the following discussion, we assume that the quantum efficiency of jots is 100%. The probability of receiving *k* photons by one jot in one frame follows a Poisson distribution:

(1) $$P\left[k\right]=\frac{e^{-H}H^{k}}{k!},$$

where *H* represents the average number of received photons.

Before ADC processing, the output signal is corrupted by root-mean-square (r.m.s.) read noise. We label the corrupted signal with *V* and the read noise with *V_n_*. By dividing *V* and *V_n_* by the conversion gain (CG), they are normalized to the number of electrons. If we label the normalized signal and the read noise with *U* and *U_n_*, respectively, then *U* = *V*/*CG* and *U_n_* = *V_n_*/*CG*. If the read noise follows a normal distribution, the probability density function (PDF) *P* [*U*, *k*] for the corrupted signal *U* and its relevant count of generated photoelectrons *k* is given by [11]:

(2) $$P\left[U,k\right]=\frac{P\left[k\right]}{U_{n}\sqrt{2\pi }}exp\left[-\frac{{\left[U-k\right]}^{2}}{2U_{n}^{2}}\right].$$

### 2.1. Single-bit QIS

**Figure 2:**
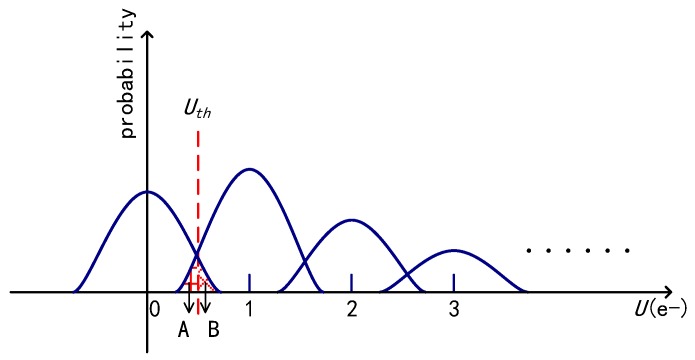
A readout signal of a single-bit quanta image sensor (QIS).

The readout signal containing shot noise and read noise in a single-bit QIS is shown in Figure 2. After processing by a 1-bit ADC, the signals that are lower than the transition point *U_th_* are quantized as “0”, or “1” otherwise. Because of the existence of read noise, bit-flip errors may occur. In [18], it is suggested that the read noise should be less than 0.30 e− r.m.s. for photoelectron counting in conventional image sensors. In a QIS, it has been proven that the read noise *U_n_* should be less than 0.15e− r.m.s to ensure a low BER, which is lower than 0.001 [12]. In fact, the read noise *U_n_* may be different among pixels in the array. In [6], 15–16% variations in read noise were observed for several kinds of specialized jots. However, in the modeling, a uniform read noise is assumed for simplicity. Based on the assumption of such a low read noise, the number of photoelectrons *k* could only be miscounted as an adjacent value *k*−1 or *k* + 1, and the probability of being miscounted as *k*-2 or *k* + 2, and further values could be neglected. For example, if the transition point *U_th_* is 0.5e− and the photoelectrons number is 1e^−^ and 2e^−^, the probabilities of miscounting *k* as 0 are 4.3 × 10^−4^ and 7.9 × 10^−24^, respectively. So, in a single-bit QIS, there are only two kinds of bit-flip errors: miscounting *k* = 1 as *k* = 0 and miscounting *k* = 0 as *k* = 1. The probability of miscounting *k* = 1 as *k* = 0, *BER* (1), is given by the area A shown in Figure 2 and is equal to:

(3) $$BER\;\left(1\right)=\int_{-\infty }^{U_{th}}P\left[U,1\right]dU.$$

The probability of miscounting *k* = 0 as *k* = 1, *BER* (0), is given by the area B and is equal to:

(4) $$BER\;\left(0\right)=\int_{U_{th}}^{+\infty }P\left[U,0\right]dU.$$

The total error rate, *BER_T_*, can be obtained by adding (3) and (4) together:

(5) $$BER_{T}=\frac{1}{2}He^{-H}erfc\left(\frac{1-U_{th}}{\sqrt{2}U_{n}}\right)+\frac{1}{2}e^{-H}erfc\left(\frac{U_{th}}{\sqrt{2}U_{n}}\right).$$

The transition points of 1-bit ADCs that are used in the same QIS are slightly different in the spatial domain. If *U_th_* follows a normal distribution in the spatial domain and the average value is 0.5e^−^, its PDF *P_uth_* [*U_th_*] is given by:

(6) $$\;\;\;P_{uth}[U_{th}]=\frac{1}{U_{thn}\sqrt{2\pi }}exp\left[-\frac{{\left(U_{th}-0.5\right)}^{2}}{2U_{thn}^{2}}\right],$$

where *U_thn_* is the standard deviation of the transition point *U_th_*. Then, the expected value of the total error rate BER is given by:

(7) $$BER=\int_{-\infty }^{+\infty }BER_{T}\cdot P_{uth}[U_{th}]dU_{th}.$$

**Figure 3:**
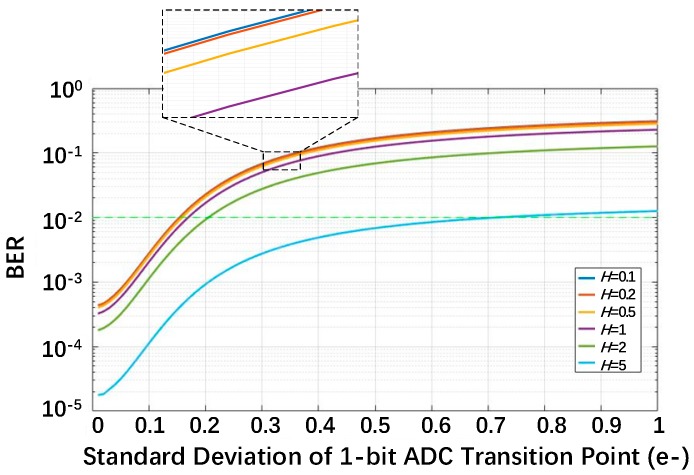
The bit error rate (BER) for a single-bit QIS as a function of the standard deviation of the transition point for different exposure levels with the read noise fixed at *U_n_* = 0.15e^−^. ADC, analog-to-digital converter.

The relationship between the BER and *U_thn_* expressed in (7) is plotted in Figure 3, and the normalized read noise *U_n_* is fixed at 0.15e^−^. The BER is higher with lower exposure, and it has a maximum value under sparse exposure (*H* ≤ 0.1). It can be explained as follows. The lower the exposure is, the larger the probabilities of *k* = 1 and *k* = 0 are, and these are the only two cases that can cause bit errors. From Figure 3, *U_thn_* should be lower than 0.15e^−^ to ensure that the BER is lower than 1% for all exposure levels. The standard deviation of the transition point in the voltage domain should be lower than 0.15e^−^ × *CG*, so a high conversion gain for a jot is essential for column or cluster ADC design. In [19], the proposed pixel achieves a high conversion gain of 220 µV/e^−^, and in [5] and [6], tapered-pump-gate jots show a 345 μV/e^−^ conversion gain on average. Anyway, the standard deviation of the transition point of 1-bit ADC should be as low as dozens of microvolts.

### 2.2. Multi-bit QIS

**Figure 4:**
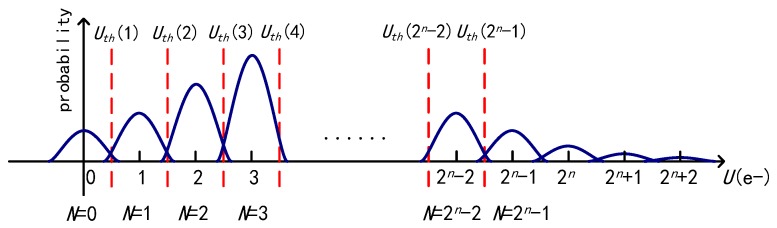
The probability distribution of the output signal and the quantization of the multi-bit QIS.

In an *n*-bit multi-bit QIS, the jot signals are quantized with 2*^n^* quantization levels, and each level corresponds to one electron as shown in Figure 4. An *n*-bit ADC has 2*^n^*−1 transition points (*U_th_*(1) to *U_th_*(2*^n^*−1)) to divide the output voltage range into 2*^n^* quantization levels, which are called bins and labeled with the number *N* in this paper. The signals corresponding to photoelectron numbers larger than 2*^n^*−1 are quantized as 2*^n^*−1.

Due to the existence of read noise, the signal corresponding to *k* photoelectrons can be quantized as other values. The miscounting probability *BER*(*k*) is the area of the probability density distribution corresponding to *k* photons spilling out of bin *N* = *k*:

(8) $$BER\left(k\right)=P\left[k\right]-\int_{U_{th}\left(k\right)}^{U_{th}\left(k+1\right)}\frac{P\left[k\right]}{U_{n}\sqrt{2\pi }}exp\left[-\frac{{\left[U-k\right]}^{2}}{2U_{n}^{2}}\right]dU.$$

Note that when *k* = 0 and *k* = 2*^n^*−1, *U_th_* (0) = −∞ and *U_th_* (2*^n^*) = +∞, respectively, in (8). In the case of *k* ≥ 2*^n^*, the bit errors could be neglected due to the small read noise. Then, the total error rate *BER_T_* is given by:

(9) $$\begin{array}{ll} BER_{T} & =\overset{2^{n}-1}{\underset{0}{\sum }}BER\left(k\right)\\ & =\frac{1}{2}\overset{2^{n}-2}{\underset{k=0}{\sum }}P\left[k\right]\cdot erfc\left[\frac{U_{th}\left(k+1\right)-k}{U_{n}\sqrt{2}}\right]+\frac{1}{2}\overset{2^{n}-1}{\underset{k=1}{\sum }}P\left[k\right]\cdot erfc\left[\frac{k-U_{th}\left(k\right)}{U_{n}\sqrt{2}}\right]. \end{array}$$

In a real image sensor, the transition point *U_th_*(*k*) of each ADC is not totally the same. In a multi-bit QIS, a pre-amplifier would be employed due to its small signal level, so all of the transition points in one ADC have the same deviation from their design values in this paper. We assume that the transition points follow a normal distribution in the spatial domain. The design value of transition point *U_th_*(*k*) is *k*−0.5. We label the standard deviation as *U_thn_*, which is normalized just as *U*, *U_n_*, etc. Then, the PDF of *U_th_*(*k*) is given by:

(10) $$P_{uth}[U_{th}\left(k\right)]=\frac{1}{U_{thn}\sqrt{2\pi }}exp\left[-\frac{{\left[U_{th}\left(k\right)-k+0.5\right]}^{2}}{2U_{thn}^{2}}\right].$$

Then, the expected value of the total error rate BER is given by:

(11) $$\begin{array}{ll} BER= & \frac{1}{2}\overset{2^{n}-2}{\underset{k=0}{\sum }}P\left[k\right]\cdot \int_{-\infty }^{+\infty }erfc\left[\frac{U_{th}\left(k+1\right)-k}{U_{n}\sqrt{2}}\right]\cdot P_{uth}[U_{th}\left(k+1\right)]d[U_{th}\left(k+1\right)]\\ & +\frac{1}{2}\overset{2^{n}-1}{\underset{k=1}{\sum }}P\left[k\right]\cdot \int_{-\infty }^{+\infty }erfc\left[\frac{k-U_{th}\left(k\right)}{U_{n}\sqrt{2}}\right]\cdot P_{uth}[U_{th}\left(k\right)]d[U_{th}\left(k\right)]. \end{array}$$

In fact, Equation (11) can also apply to the case of *n* = 1.

**Figure 5:**
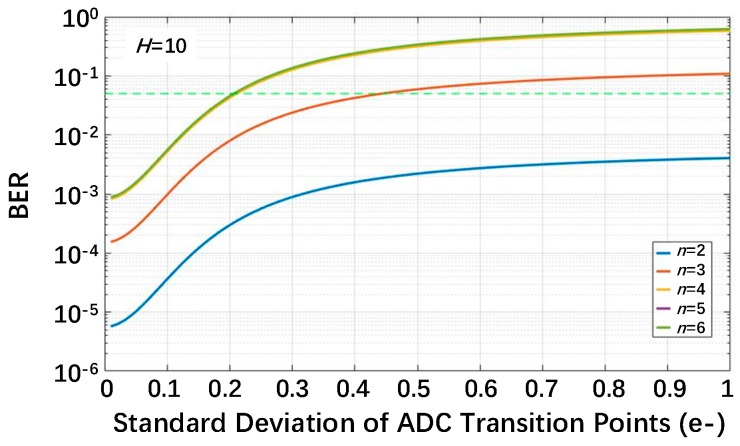
The BER as a function of standard deviation *U_thn_* for different QIS bit depths for exposure *H*=10 and read noise *U_n_* = 0.15e^−^.

Figure 5 shows the relationship between the BER and the standard deviation *U_thn_* for different bit depths of the QIS, and the BER becomes larger with an increasing *U_thn_*. The dashed line represents a constant 5% reference. Because a bit error has a smaller impact on a multi-bit QIS than a single-bit one, a higher reference value is set. As a conclusion, the BER performance is better in the multi-bit QIS with a higher bit depth. As shown in Figure 4, because of the symmetry of the normal distribution, the probability distribution of the signal corresponding to *k* photoelectrons spills out into bins *N* > *k* and bins *N* < *k* for 1 ≤ *k* ≤ 2*^n^*−2. For *k* = 0 and *k* = 2*^n^*−1, their distributions could only spill out into bins on one side, so less bit errors occur. For *k* ≥ 2*^n^*, the signals hardly cause bit errors. When the exposure *H* is fixed, the probability of *k* ≥ 2*^n^*−1 decreases with an increasing bit depth *n*, which also leads to an increasing BER.

**Figure 6:**
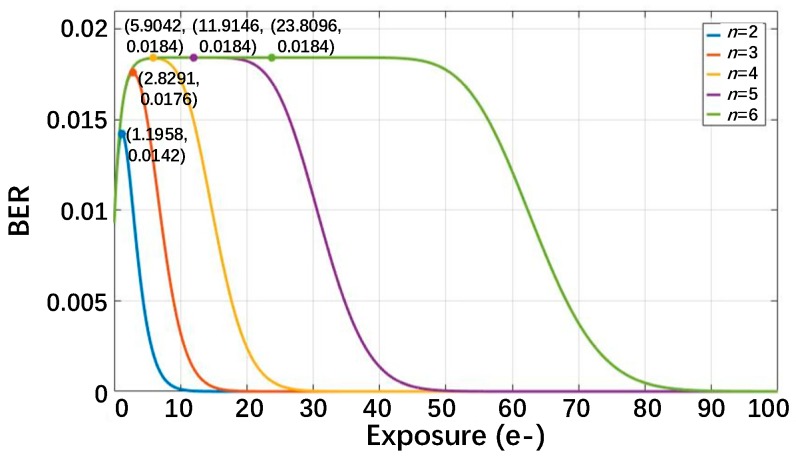
The BER as a function of the exposure *H* for different QIS bit depths for *U_n_* = 0.15e^−^ and *U_thn_* = 0.15e^−^. The extreme points are marked.

Furthermore, with an increasing exposure *H* for a fixed bit depth, the probability distribution of the signals would go through three stages: (1) most signals distribute in bin *N* = 0; (2) most signals distribute in inner bins; and (3) most signals distribute in bins *N* ≥ 2*^n^*−1. Accordingly, as shown in Figure 6, the BER would firstly increase, then decrease, and, finally, approach zero, so there are extreme points for those plots. However, high exposure with a BER close to zero is not expected to occur in the application of a QIS, because it means that the photon number would go beyond the quantization range and the QIS would not be able to execute the photon counting function. In Figure 6, it can also be seen that when the bit depth *n* is higher (*n* = 5 or 6), the middle part of the curves is almost flat. The reason is that the number of photoelectrons *k* with the highest probability is far away from both bin *N* = 0 and bin *N* = 2*^n^*−1, so a little higher or lower exposure would not change the situation that bin *N* = 0 and bins *N* ≥ 2*^n^*−1 collect few photoelectrons.

The extreme points of the different curves are also shown in Figure 6. The *x*-axis value of an extreme point represents the “worst” exposure, where the highest BER is obtained. Equation (11) could be written as

(12) $$\begin{array}{ll} BER= & \frac{1}{2}\overset{2^{n}-1}{\underset{k=1}{\sum }}P\left[k-1\right]\cdot \int_{-\infty }^{+\infty }erfc\left[\frac{U_{th}\left(k\right)-k+1}{U_{n}\sqrt{2}}\right]\cdot P_{uth}[U_{th}\left(k\right)]d[U_{th}\left(k\right)]\\ & +\frac{1}{2}\overset{2^{n}-1}{\underset{k=1}{\sum }}P\left[k\right]\cdot \int_{-\infty }^{+\infty }erfc\left[\frac{k-U_{th}\left(k\right)}{U_{n}\sqrt{2}}\right]\cdot P_{uth}[U_{th}\left(k\right)]d[U_{th}\left(k\right)]. \end{array}$$

**Figure 7:**
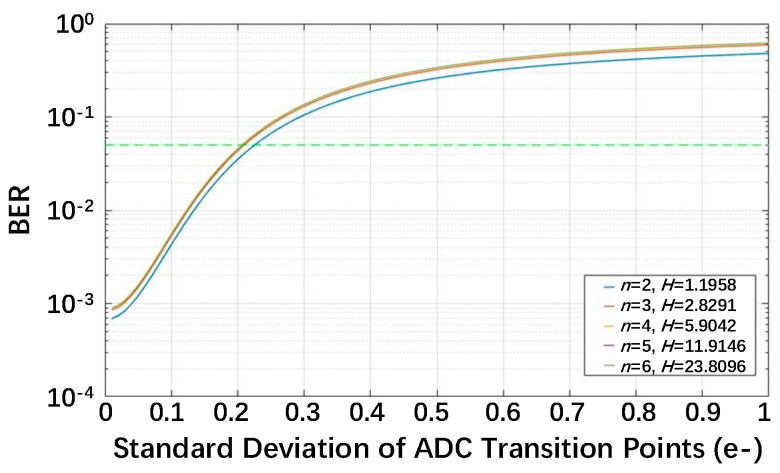
The BER as a function of *U_thn_* for different QIS bit depths *n* and their relevant worst exposure for read noise *U_n_* = 0.15e^−^.

In (12), the expectation of the normal distribution function *P_uth_*[*U_th_*(*k*)] is *k*−0.5. Meanwhile, the two expressions *U_th_*(*k*) – *k* + 1 and *k* − *U_th_*(*k*) have a symmetry relation and the symmetry axis is *k*−0.5, so the two integral terms in (12) are equal. All of the transition points have the same standard deviation *U_thn_*, so the values of the two integral terms will not change with *k*. So, (12) is equal to:

(13) $$BER=\frac{1}{2}\int_{-\infty }^{+\infty }erfc\left[\frac{U_{th}\left(1\right)}{U_{n}\sqrt{2}}\right]\cdot P_{uth}[U_{th}\left(1\right)]d[U_{th}\left(1\right)]\cdot \overset{2^{n}-1}{\underset{k=1}{\sum }}\left(P\left[k\right]+P\left[k-1\right]\right),$$

where *k* = 1 in the integral terms. In (13), the BER is the product of an integral term and an accumulation term. The integral term is independent of the variable *H*, so the calculation for the worst exposure is only dependent on the accumulation term, which does not contain the variable *U_thn_*. As a result, the worst exposure is independent of *U_thn_*. To determine the impact of *U_thn_* on the BER, the worst exposure should be used. In Figure 7, the relationship between the BER and *U_thn_* for different QIS bit depths *n* with their respective worst exposure is plotted. To ensure that the BER is lower than 5%, *U_thn_* should be lower than 0.22e- for a bit depth *n* = 2 and 0.21e^−^ for *n* = 3–6. In the following discussion, 0.21e^−^ is chosen for all bit depths in the multi-bit QIS.

In fact, the read noise in jots of the QIS may be other values. As a result, the tolerance for the mismatch strength of the ADC transition points could be different. For example, in a single-bit QIS in which the BER is lower than 1%, *U_thn_* should be lower than 0.19e- and 0.08e^−^ when the read noise is 0.1e^−^ and 0.2e^−^, respectively. In a multi-bit QIS in which the BER is lower than 5%, *U_thn_* should be lower than 0.24e^−^and 0.16e^−^ when the read noise is 0.1e^−^ and 0.2e^−^, respectively. Furthermore, BER values of 1% and 5% are only for reference, and could be adjusted in the design of a real sensor.

## 3. Imaging Models for the Effect of the Transition Points Mismatch

### 3.1. Single-bit QIS

**Figure 8:**
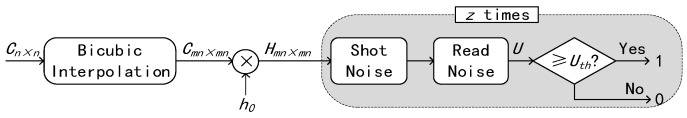
A block diagram of the single-bit QIS imaging model.

**Figure 9:**
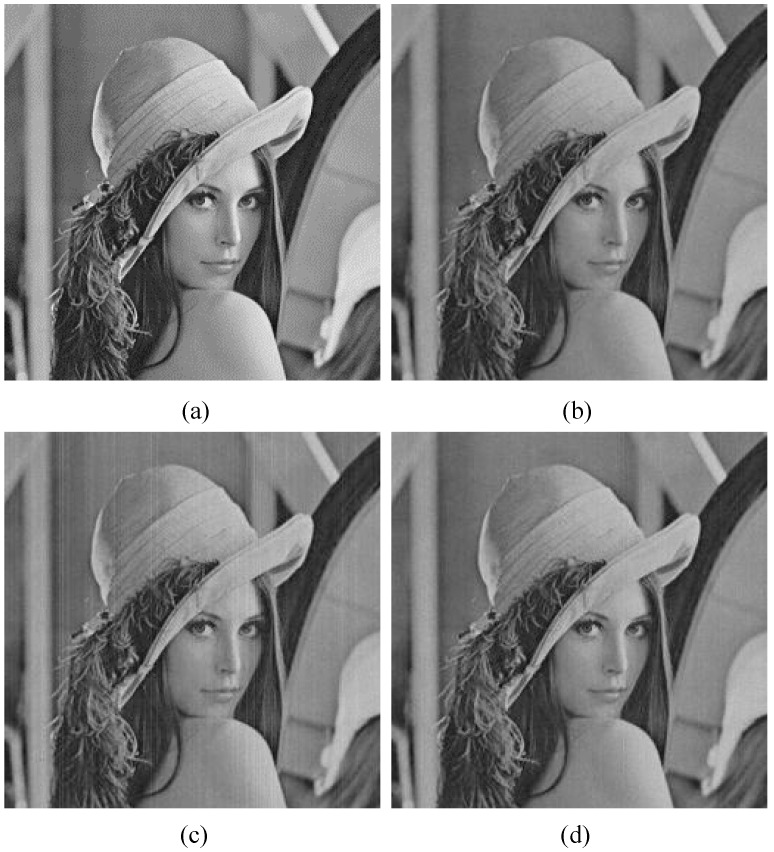
(**a**) The standard 256 × 256 image “lena”; (**b**) a reconstructed image contaminated by shot noise and read noise (*U_n_* = 0.15e^−^) without the mismatch of a 1-bit ADC transition point (*U_th_* = 0.5) for *m* = 16 and *z* = 16, and the grayscale similarity index (GSI) is 0 LSB; (**c**) a reconstructed image with a transition point mismatch, *U_thn_* = 0.15e- added on the basis of (**b**), and the GSI is 3.31 LSB; (**d**) a reconstructed image from the same condition as (**c**), except that *m* = 64, *z* = 1, and the GSI is 1.74 LSB.

The imaging model for the single-bit QIS employs the method described in [10] as shown in Figure 8. The grayscale matrix *C_n × n_* of a standard 256 × 256 image (“lena”) as shown in Figure 9a is abstracted, and every element is converted to an *m* × *m* two-dimensional matrix through bicubic interpolation. The grayscale values (0-255) cannot be used as the exposure levels because they do not match with the dynamic range of a single-bit QIS, so the enlarged matrix is multiplied by an illumination factor *h_0_* that is smaller than 1, and each new element is the average exposure level *H* for a jot as well as its ideal output signal expressed as an electron number. Each exposure *H* is converted to a random number according to a Poisson distribution. Then, the integer is converted to a random number according to a normal distribution with a standard deviation *U_n_*. These two conversions simulate shot noise and read noise, respectively. In the quantization process, the output signal containing shot noise and read noise is compared to a transition point *U_th_* and a binary jot bit is obtained. The variable *U_th_* could be a constant value for the whole jot array for ideal 1-bit ADCs or vary from column to column for actual 1-bit ADCs when ADCs are column parallel. Repeat the operations on the exposure *H* for *z* times to generate *z* captured sequential fields. Thus, the original value of a pixel is converted to an *m* × *m* × *z* binary jot bit cubicle. In the image reconstruction process, the bit data of a cubicle are added together, and the result represents the gray level of a pixel in the final image. The sum of a cubicle has to be multiplied by 255/*m*^2^*z* to match with the grayscale range of the original image. In this imaging model, the spatial oversampling factor is *m*^2^, the temporal oversampling factor is *z*, and the equivalent FWC of the final image is *m*^2^*z*.

The BER is not suitable for comparing reconstructed images with different spatial oversampling factors or bit depths. This is explained in later discussion. Instead, this paper employs an evaluation index, called the grayscale similarity index (GSI), to calculate the difference between a reconstructed image and a reference image. It can be expressed as:

(14) $$GSI=\sqrt{\frac{1}{256\times 256}\overset{256}{\underset{i=1}{\sum }}\overset{256}{\underset{j=1}{\sum }}{\left[c_{rec}\left(i,j\right)-c_{ref}\left(i,j\right)\right]}^{2}},$$

where *c_rec_*(*i*, *j*) and *c_ref_*(*i*, *j*) is the grayscale value of pixel (*i*, *j*) in the reconstructed image and the reference image. The reference image is a reconstructed image with ideal ADCs in the models.

In the following discussion, the equivalent FWC is *m*^2^*z* = 4096, and the illumination factor *h_0_* = 1/400 to guarantee that the average exposure level *H* of each jot is smaller than full exposure *H* = 1. In fact, *h_0_* = 1/255 is enough and is compatible with that in the multi-bit case. However, in the simulation, it was found that the reconstructed image with a little smaller *h_0_* has better contrast. The reference image is reconstructed with a constant transition point *U_th_* = 0.5 for *m* = 16 as shown in Figure 9b. If different values of *m* and *z* are used while the value of *m*^2^*z* is fixed, the imaging quality would not change because the shot noise and the read noise are random in both the spatial and temporal domains. Then, the mismatch of transition points is introduced into column parallel 1-bit ADCs and the standard deviation *U_thn_* is 0.15e-, which is suggested in Section 2. The reconstructed image is shown in Figure 9c, and its GSI is 3.31 LSB. Compared to Figure 9b, Figure 9c shows obvious stripes. The white stripes are caused by column 1-bit ADCs with lower transition point values, and the dark stripes are caused by 1-bit ADCs with higher transition point values. Figure 9d illustrates a better image with few stripes, and its GSI is 1.74 LSB. It is reconstructed from the same conditions as Figure 9c, except that *m* = 64 and *z* = 1. Other GSI values for different spatial oversampling factors are summarized in Table 1. Different from shot noise and read noise, the mismatch of ADC transition points is actually a kind of noise in the spatial domain. In the same cubicle, a false positive count and a false negative count could cancel each other. A larger *m* value means a more sufficient cancellation, and also means that a bit error in a cubicle has a smaller impact on the final image. That could explain why a larger spatial oversampling factor is beneficial to the image quality, and why the BER is not suitable for comparing reconstructed images with different spatial oversampling factors. 

**Figure 10:**
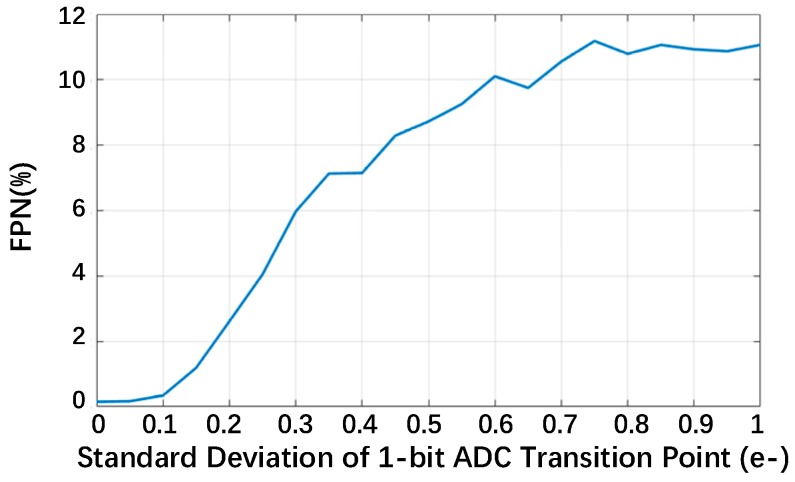
The fixed-pattern noise (FPN) of reconstructed images with *m* = 64 and *z* = 1.

In Figure 9, it is observed that the transition point mismatch causes column stripes in the reconstructed image, which is actually a kind of fixed-pattern noise (FPN). To clearly understand the relationship between the transition point mismatch and the FPN, a 256 × 256 image with a uniform gray level of 128 is used in place of the “lena” image in the imaging model. In the model, *m* = 64 and *z* = 1. We calculate the mean gray level of every column in the reconstructed image, then the standard deviation of this group of mean values is divided by their average. The result is used to represent the FPN. Figure 10 shows the FPN of reconstructed images with different standard deviations of the transition point, and it proves that the FPN increases with a stronger transition point mismatch.

### 3.2. Multi-bit QIS

The imaging model of the multi-bit QIS is established based on that of the single-bit QIS. The illumination factor *h_0_* here is (2*^n^*−1)/255. In the quantization process, the signal of a jot is compared to a group of transition points (*U_th_* (1) to *U_th_* (2*^n^*−1)) to obtain an *n* bits number. All columns of jot signals could be compared to the same group of transition points for ideal ADCs or a different group of transition points for non-ideal ADCs. Note that 2*^n^*·*m*^2^*z* is 4096; however, it is not the equivalent FWC. In fact, the equivalent FWC is (2*^n^*−1)·*m*^2^*z*, which is a little smaller than 4096. In the image reconstruction process, the sum of a jot data cubicle is multiplied by 255/(2*^n^*·*m*^2^*z*). In the discussion about the mismatch of ADC transition points, a pre-amplifier is expected to be used before an ADC due to the extremely small signals (e.g., maximally about 10 millivolts for a bit depth *n* = 4), so the 2*^n^*−1 transition points of an ADC have the same deviation from their ideal values.

**Figure 11:**
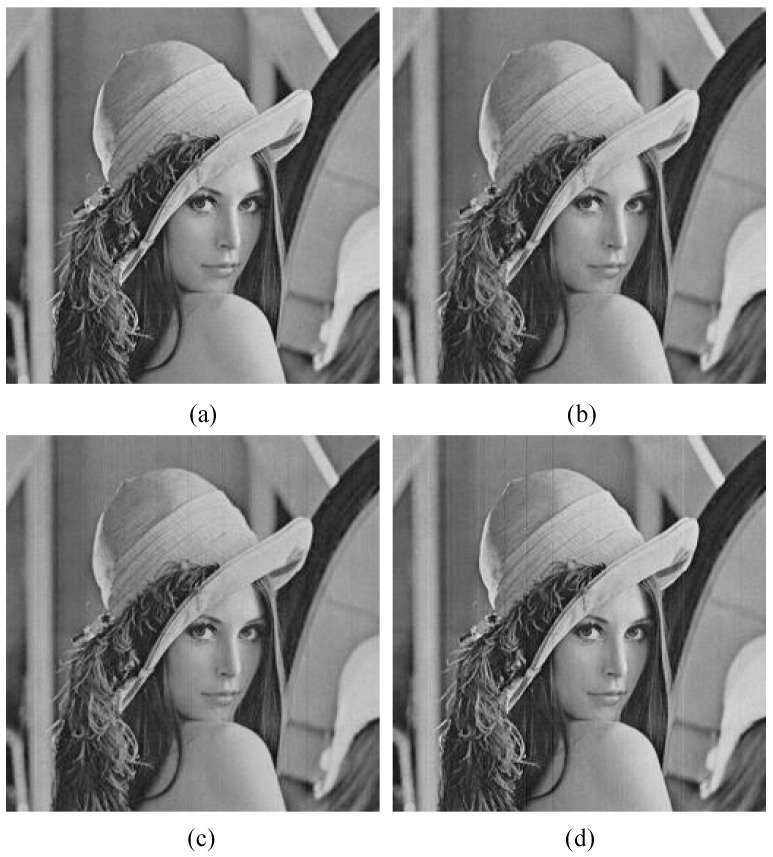
(**a**) A reconstructed image contaminated by shot noise and read noise (*U_n_* = 0.15e^−^) without a mismatch of ADC transition points (*U_t_*_h_ (*k*) = *k*-0.5) for *m* = 4, *z* = 16, and *n* = 4, and the GSI is 0 LSB; (**b**) a reconstructed image with a mismatch of ADC transition points (*U_thn_* = 0.21) added on the basis of (a), and the GSI = 1.13 LSB; (**c**) *m* = 4, *z* = 32, *n* = 3, and the GSI = 1.93 LSB; (**d**) *m* = 1, *z* = 256, *n* = 4, and the GSI = 2.06 LSB.

The reference image is reconstructed with ideal ADC transition points *U_th_*(*k*) = *k*-0.5 for *m* = 4, *z* = 16, and an ADC bit depth *n* = 4 as shown in Figure 11a. In Figure 11b, the mismatch of transition points with standard deviation *U_thn_* = 0.21, which is suggested in Section 2, is introduced into the quantization process, and its GSI is 1.13 LSB. Compared to Figure 9c, there are no obvious stripes even though the *m* value is smaller. The reason is that there are only two jot states in the single-bit QIS and a bit error means a totally opposite result; however, in the multi-bit QIS, for example, quantizing eight photoelectrons in a jot as nine photoelectrons has a very small impact on a four-bit QIS with 16 states in total. That could also explain why the BER could not be used to compare reconstructed images with different bit depths. Figure 11c is a reconstructed image for a lower bit depth, and some slight stripes can be observed. The GSI of this image is 1.93 LSB. It proves that, among the multi-bit QISs with different bit depths, the one with the highest bit depth has the highest tolerance for the mismatch of ADC transition points. Just like the single-bit QIS, the spatial oversampling factor in the multi-bit QIS influences the image quality. Figure 11d shows an image reconstructed from the same condition as Figure 11b, except that *m* = 1 and *z* = 256. Its GSI is 2.06 LSB, and slight stripes can be observed. The GSI values for different bit depths and spatial oversampling factors are summarized in Table 2.

The design value of the transition point *U_th_* is 0.5 in the single-bit QIS and *U_th_*(*k*) is *k*−0.5 in the multi-bit QIS here; however, it could be adjusted to the global exposure level (controlled by the illumination factor *h_0_* in the models) to achieve a lower BER [12]. So, in a practical application, the QIS may obtain better images with the same transition points mismatch conditions that were simulated in the former discussion through adjusting the design values of the ADC transition points.

**Figure 12:**
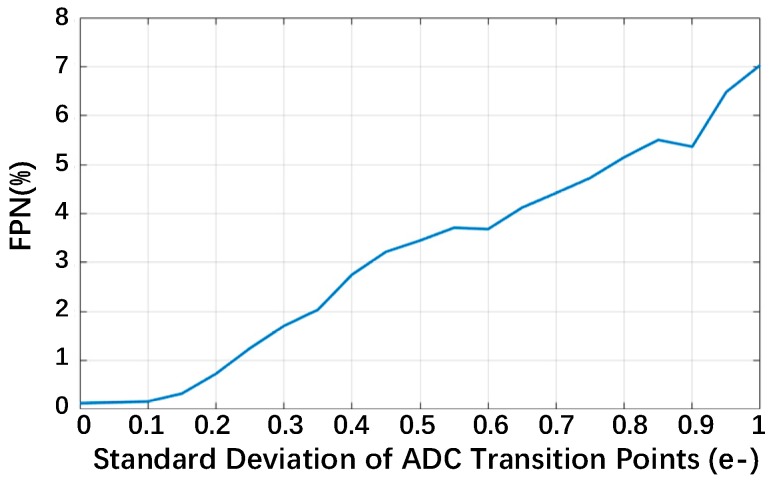
The FPN of reconstructed images with *m* = 4, *z* = 16, and *n* = 4.

Similarly, the relationship of the FPN of the reconstructed images and the standard deviations of the transition points of the multi-bit QIS is plotted in Figure 12 with *m* = 4, *z* = 16, and *n* = 4. It also proves that the FPN increases with a stronger transition point mismatch. Usually, the FPN could be calibrated through image-processing techniques; however, this is not suggested in a QIS. A QIS is actually a photon counting system and needs to record the temporal and spatial arrival information of photons as much as possible. Image-processing techniques could smooth the image but lead to arrival information loss and distortion; so, realizing a small transition points mismatch through circuit design is a better choice.

## 4. Conclusions

The effect of an ADC transition points mismatch on a QIS is discussed, and related mathematical models are established. The BER is higher when the mismatch of 1-bit and *n*-bit ADC transition points is stronger. The tolerance for the mismatch is determined to ensure a low BER and good image quality. The standard deviation of the 1-bit ADC transition point should be less than 0.15e^−^ if the reference value of the BER is 1% for a single-bit QIS; the standard deviation of *n*-bit ADC transition points should be less than 0.21e^−^ if the reference value of the BER is 5% for a multi-bit QIS.

Imaging models for a QIS are established to observe the imaging quality when a low BER is guaranteed. The models prove that, while the image quality is affected by the mismatch of 1-bit and *n*-bit ADC transition points, a higher bit depth of a multi-bit QIS and a larger spatial oversampling factor could reduce its effect. The imaging models also prove that the FPN increases with a stronger transition point mismatch.

## Figures and Tables

**Table 1:** The GSI values for different spatial oversampling factors with *U_thn_* = 0.15.

*m* ^2^	1^2^	2^2^	4^2^	8^2^	16^2^	32^2^	64^2^
GSI (LSB)	9.71	8.44	5.28	4.30	3.31	2.11	1.74

**Table 2:** The GSI values for different bit depths and spatial oversampling factors with *U_thn_* = 0.21.

	*m* ^2^	1^2^	2^2^	4^2^	8^2^	16^2^	32^2^
GSI (LSB)	2-bit	9.51	5.18	4.00	2.98	2.18	1.56
	3-bit	3.87	2.70	1.93	1.45	1.16	-
	4-bit	2.06	1.51	1.13	0.76	0.68	-
	5-bit	1.15	0.89	0.59	0.54	-	-
	6-bit	0.62	0.47	0.42	0.37	-	-
